# 

**Published:** 1895-10-05

**Authors:** 


ABSOLUTELY PURE, therefore BEST.

**CADBURY'S COCOA**

Of absolute purity and freedom from alkali." —*Braithwaite.*

**CADBURY'S COCOA**

"The Typical Cocoa of English Manufacture. Absolutely Pure."—*The Analyst.*

October 5th, 1895.

### THE HOSPITAL

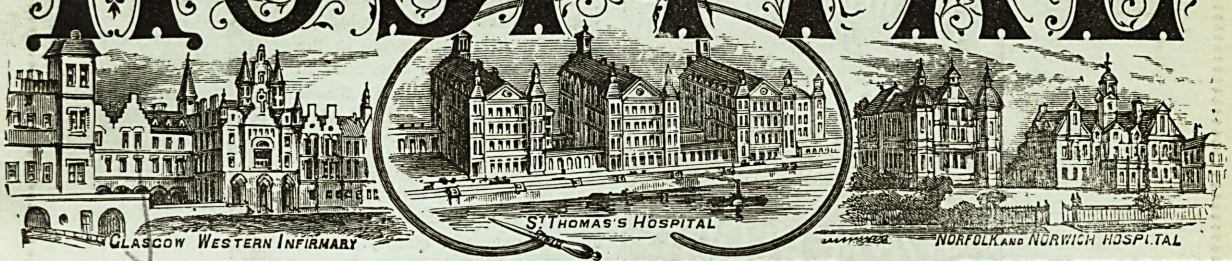

No. 471. Vol. XIX.
Saturday, Oct. 5th, 1895.
With Index to Vol. XVIII.

WITH EXTRA NURSING SUPPLEMENT.
[Registered at the General Post Office as a Newspaper for transmission in the United Kingdom and Abroad.]

PRICE TWOPENCE.

Per Annum, 10s. 6d., post free.

Monthly Parts, 6d.; post free, 8d.

Ditto per Annum, 8s.6d., post free.

